# Carrier screening for Alport syndrome: The clinical importance of heterozygosity for pathogenic or likely pathogenetic variants

**DOI:** 10.1002/jgc4.70045

**Published:** 2025-05-02

**Authors:** Vivienne Souter, Lisa Johnson, Emily Becraft, Ashley Cantu‐Weinstein, Hossein Tabriziani, Peter Benn, Clifford E. Kashtan

**Affiliations:** ^1^ Natera, Inc. Austin Texas USA; ^2^ UCONN Health Farmington Connecticut USA; ^3^ University of Minnesota Medical School Minneapolis Minnesota USA

**Keywords:** Alport syndrome, carrier testing, *COL4A3‐5*‐related disease, genetic testing, heterozygous, obstetrics, prenatal, referral practices, risk management

## Abstract

Reproductive carrier screening aims to identify individuals at an increased chance of having children affected by genetic conditions. However, testing can also reveal health implications for autosomal or X‐chromosome heterozygotes. One such example is screening for Alport syndrome (*COL4A3‐5*‐related disease) which is one of the most common causes of inherited chronic kidney disease. Alport syndrome heterozygotes have an increased chance for chronic kidney disease. Monitoring and providing early treatment can slow kidney disease progression and delay the onset of kidney failure. We provide information on Alport syndrome and propose a simple management algorithm for individuals found on carrier screening to have a pathogenic or likely pathogenic variant in one or more of the Alport syndrome genes. We emphasize the importance of genetic counseling, partner screening, and cascade testing to identify at‐risk family members, including existing children. Clinical management includes baseline evaluation for kidney disease, nephrology referral when needed, enhanced pregnancy surveillance for proteinuria and hypertension, and long‐term follow‐up. The proposed management plan serves as an example for other conditions where screening identifies heterozygotes with a variable chance for disease in the individual tested.

**What is known about this topic**

Emerging evidence suggests heterozygosity for certain autosomal recessive or X‐linked genetic conditions on carrier screening panels has clinical implications beyond the chance of having an affected child. Alport syndrome is one of these conditions where heterozygosity for a pathogenic or likely pathogenic variant is relatively common, and these individuals may have Alport syndrome or related kidney complications.

**What this paper adds to the topic**

We present a simple algorithm for the management of individuals identified as carriers of pathogenic/likely pathogenic Alport syndrome gene variants. We consider reproductive genetic counseling issues, additional testing, and future surveillance indicated for these heterozygotes.

## 1 INTRODUCTION

The primary purpose of reproductive carrier screening is to identify people with an increased chance of having a child affected by specific genetic conditions (Committee Opinion No. 691: Carrier screening for genetic conditions, 2017; Gregg et al., 2021; Sagaser et al., 2023). Current recommendations for carrier screening differ between US professional societies, but it is agreed that information about carrier screening should be provided to all pregnant people or, ideally, prior to pregnancy (Committee Opinion No. 691: Carrier screening for genetic conditions, 2017; Gregg et al., 2021; Sagaser et al., 2023). Recognizing the limitations of race or ethnic‐specific carrier screening, carrier screening utilizes a pan‐ethnic approach. Moreover, inexpensive genetic technologies have facilitated carrier screening for much larger numbers of genetic conditions (Gregg et al., 2021).

Reproductive carrier screening is generally performed on apparently healthy individuals. However, there is increasing recognition that for some genetic conditions, heterozygotes themselves may also have an increased chance of symptoms, including complications during pregnancy (e.g., cardiomyopathy for dystrophinopathy carriers, hyperammonemia for ornithine transcarbamylase deficiency carriers) (Clevenger et al., 2023; Gbur et al., 2021; Reiner et al., 2023; Strauss et al., 2023). Since carrier screening occurs predominantly in the prenatal setting, obstetric professionals and prenatal genetic counselors should be aware of the implications for individuals with heterozygous variants, the opportunity for preventative interventions, and also the psychosocial aspects of learning about potential health implications.

This commentary provides information about Alport syndrome heterozygotes and presents a simple algorithm for surveillance and management based on current knowledge about the condition. We briefly review the genetic basis of Alport syndrome, discuss genetic counseling issues associated with carrier identification, and present potential pathways for the evaluation and management of people found to have a pathogenic/likely pathogenic (P/LP) variant in *COL4A3, COL4A4*, or *COL4A5* on carrier screening.

## 2 SUMMARY OF ALPORT SYNDROME

Alport syndrome is an important genetic cause of chronic kidney disease and kidney failure that can arise from pathogenic variants in any of three genes – *COL4A3, COL4A4, and COL4A5* – which together encode the collagen IV alpha345 scaffolds of basement membranes in the kidney, cochlea, and eye (Kashtan, 2017). Pathogenic variants in *COL4A3* and *COL4A4* cause autosomal forms of Alport syndrome (autosomal dominant or recessive), while pathogenic variants in *COL4A5* cause X‐linked Alport syndrome (Kashtan, 2017). Individuals with Alport syndrome exhibit hematuria, often accompanied by proteinuria and decreased kidney function, and may also display sensorineural deafness and characteristic ocular abnormalities.

The precise prevalence of Alport syndrome in the US is uncertain, but estimates of the prevalence of heterozygous pathogenic variants in these genes are ~1 in 100 individuals for either *COL4A3* or *COL4A4*, and ~1 in 2000 individuals for hemizygous or heterozygous variants in *COL4A5* (Gibson et al., 2021). In 46, XX individuals with heterozygous variants in *COL4A5*, the chance of kidney failure by age 60 years is 15%–30% (Savige et al., 2021). For individuals with heterozygous variants in *COL4A3* and *COL4A4*, the available evidence suggests a 13%–15% chance of end‐stage renal disease (Matthaiou et al., 2020).

Alport syndrome individuals are at an increased chance for pregnancy complications, including preeclampsia and proteinuria (Brunini et al., 2018; Gosselink et al., 2024). In the largest study of pregnant individuals with Alport syndrome (Gosselink et al., 2024), the ALPART network (mAternaL and fetal PregnAncy outcomes of women with AlpoRT syndrome) reported retrospective outcomes for 192 singleton pregnancies ≥20 weeks' gestation in 116 people with Alport syndrome, 78% of whom had early kidney disease. For pregnancies with outcomes, 23% had gestational hypertension, 20% preeclampsia, 17% preterm birth (<37 weeks), and 76% had new onset or doubling of proteinuria. There was no evidence of a detrimental effect of pregnancy on kidney function. This study included pregnant people with a pre‐existing diagnosis of Alport syndrome, which is a different population from those identified as heterozygotes in carrier screening. Additional research is needed to elucidate pregnancy outcomes in those identified by carrier screening.

Early diagnosis of Alport syndrome is key to improving long‐term outcomes. Appropriate treatment in non‐pregnant people with inhibitors of the renin–angiotensin–aldosterone system (e.g., angiotensin‐converting enzyme inhibitors, angiotensin receptor blockers) can substantially slow the progression of kidney disease and delay kidney failure by many years (Alshahrani, 2023). The availability of therapy, which is most effective when initiated while kidney function is normal (Gross et al., 2012), highlights the importance of evaluation and follow‐up of people who exhibit a pathogenic variant in *COL4A3, COL4A4*, or *COL4A5*.

As reproductive carrier screening becomes more widely utilized, substantial numbers of individuals will likely be found to have a P/LP variant in *COL4A3, COL4A4*, or *COL4A5*. Of 91,000 pregnant people screened by a commercial laboratory, the observed carrier frequency for a P/LP variant was one in 279 for *COL4A3*, one in 332 for *COL4A4*, and one in 3055 for *COL4A5*, giving an overall carrier rate of one in 144 for any one of the Alport syndrome genes (Souter et al., 2023).

## 3 GENETIC COUNSELING FOR HETEROZYGOTES

Reproductive carrier screening needs informed consent, which should include counseling regarding the possibility of unexpected or uncertain results, such as information about personal health for the individual undergoing screening. This can include information on variably penetrant or expressed conditions and identification of late‐onset conditions that could be present in the individual tested, their children, or other family members. As a practical matter, it is not possible to present detailed information for all disorders in broad panels, and therefore disease‐specific information is often only provided following a positive test. However, it should be recognized that the information could be viewed as beneficial or stigmatizing, creating uncertainty or fear of illness.

Health professionals requesting carrier screening may be unfamiliar with Alport syndrome, the potential for chronic kidney disease in individuals who are heterozygous for a P/LP variant in one of the Alport syndrome genes, appropriate management and surveillance, or the potential for preventative treatment outside of pregnancy. Informing heterozygotes of the significance of screening results and options for additional testing is often placed on genetic counselors, who need to be familiar with resources for variant interpretation and follow‐up testing options. There is a paucity of data on clinical outcomes for Alport syndrome P/LP heterozygotes identified through carrier screening. However, contemporary counseling often requires communication of the chance for disease based on limited experience for the specific genetic variant and disorders (such as Alport syndrome) where there appears to be considerable variability in penetrance and expressivity.

Alternative strategies to identify affected pregnancies or children exist that do not report heterozygotes. For instance, the Australian Reproductive Genetic Carrier Screening Project (Mackenzie's Mission) provided nearly 10,000 couples with carrier screening. Reproductive partners were screened together to maximize the prediction for transmission of hereditary conditions to their children, and individual carrier status was not reported to participants (Kirk et al., 2021). While maternal carrier status for an X‐linked Alport syndrome gene variant (*COL4A5*) was disclosed, P/LP variants in *COL4A3* or *COL4A4* were not reported unless both partners had at least one variant (Kirk et al., 2021). Similarly, the Generation Study in the UK offers expanded newborn screening for ~200 genetic conditions but does not report heterozygous carrier status (Genomics England, 2024). The conditions included meet the following criteria: (1) usually appear in the first few years of life; (2) can be improved if caught early; and (3) can be treated through the National Health Service in England (Genomics England, 2024). Heterozygous carrier status for an Alport syndrome P/LP variant would satisfy these criteria, but the study only reports results for newborns with homozygous or hemizygous X‐linked P/LP variants. Strategies that exclude the reporting of individual carrier status miss opportunities for education regarding carrier conditions such as Alport syndrome and reduce opportunities to identify heterozygotes in other family members.

The challenge of counseling individuals who are carriers for a disorder that can be expressed in heterozygotes is not confined to Alport syndrome (Clevenger et al., 2023; Gbur et al., 2021; Reiner et al., 2023; Strauss et al., 2023). Heterozygosity is often associated with a milder phenotype or later onset than those present in homozygous individuals (Clevenger et al., 2023; Gbur et al., 2021; Reiner et al., 2023; Strauss et al., 2023). However, similar to Alport syndrome, in some cases, this can provide actionable health information, for example, the presence of familial hypercholesterolemia P/LP gene variants (Souter et al., 2024). Additionally, it might provide information relevant to pregnancy, which is when most people undergo reproductive carrier screening. A recent publication identified 12 genes, including the three genes associated with Alport syndrome (*COL4A3, COL4A4, COL4A5*), on a 274‐gene panel with the potential for carrier manifestations during pregnancy (Souter et al., 2023). The interdisciplinary nature and required specialized knowledge of genomics make genetic counseling of key importance in the optimal management of these positive carrier test results.

## 4 CLINICAL RECOMMENDATIONS FOR THE EVALUATION AND MANAGEMENT OF PREGNANT INDIVIDUALS HETEROZYGOUS FOR ALPORT SYNDROME P/LP VARIANTS

**FIGURE 1:**
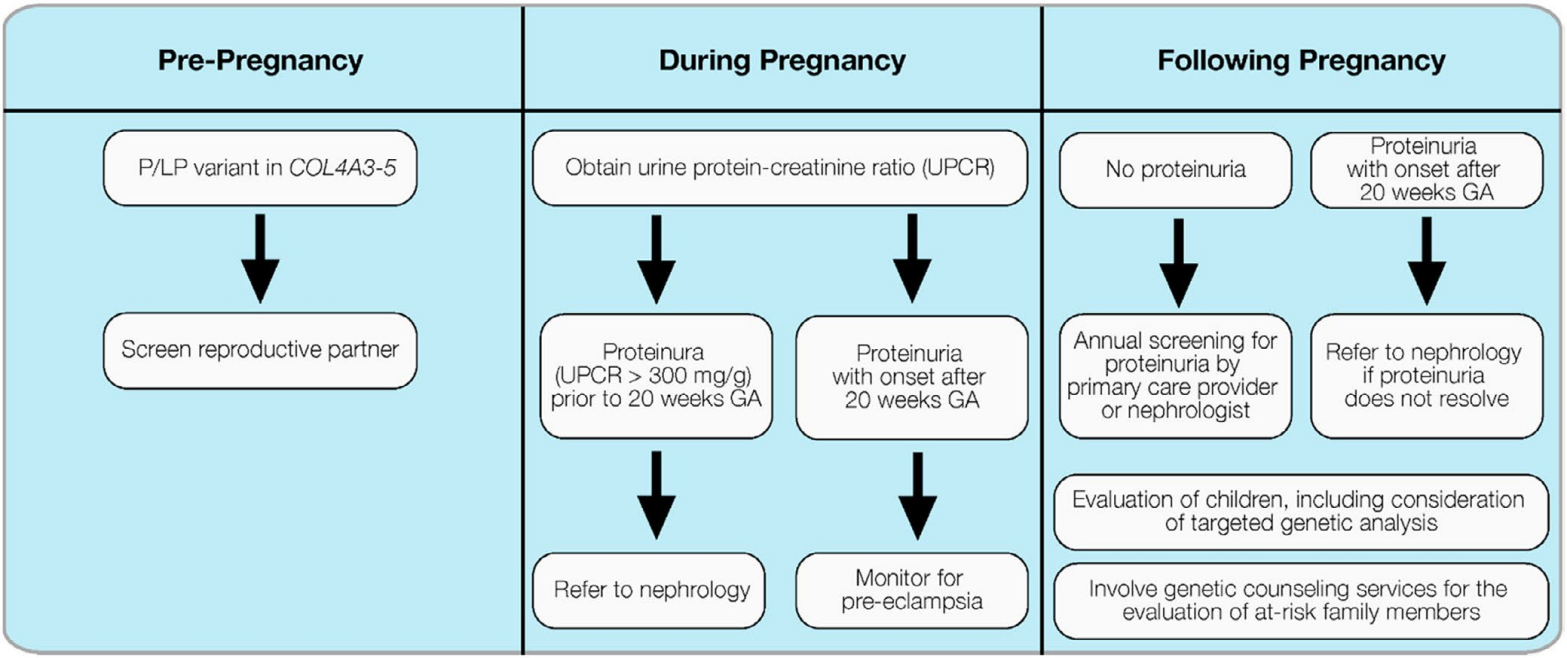
Potential management algorithm for individuals with positive carrier screenings for Alport syndrome. GA, gestation; P/LP, pathogenic/likely pathogenic.

Although there are recommendations available for the clinical management of adults with Alport P/LP variants, (Nozu et al., 1993–2025) there are currently no published recommendations specifically for the evaluation and management of the pregnant person with an Alport syndrome P/LP gene variant. We present the following recommendations for evaluating and managing these individuals during and after pregnancy, as well as for evaluating and managing the pregnant person's family and the child or children resulting from the pregnancy; these recommendations are summarized in Figure 1. We recognize that individual circumstances, such as access to nephrologists and genetic counselors and insurance coverage limitations, may cause variation in implementing these recommendations.

### 4.1 Management of individuals identified with P/LP variants in the obstetric setting

Proteinuria is a key risk factor for kidney disease progression in people with a *COL4A3–5* variant, and in a pregnant person may be associated with an increased chance for preeclampsia and development of nephrotic syndrome levels of proteinuria. Screening for proteinuria by urine protein–creatinine ratio (UPCR) should ideally occur before pregnancy or at least before 20 weeks gestation. Proteinuria (UPCR >0.3 mg/mg or 300 mg/g) present before pregnancy or before 20 weeks gestation indicates the presence of kidney disease, while proteinuria appearing after 20 weeks gestation could be due to Alport syndrome‐associated, new onset proteinuria or preeclampsia. Pregnant persons with new onset proteinuria should be evaluated and followed for hypertension, and kidney function should be evaluated through serum creatinine measurements. When proteinuria is present before pregnancy or 20 weeks' gestation, the patient should be referred to nephrology and managed using existing clinical practice guidelines for kidney disease during pregnancy (Fitzpatrick et al., 2016; Schmidt et al., 2022; Wiles et al., 2019). Prophylactic low‐dose aspirin should also be considered for prevention of preeclampsia (Rolnik et al., 2017).

### 4.2 Management of individuals with P/LP variants outside of pregnancy

In the absence of proteinuria, annual screening for onset of proteinuria by a primary care provider or a nephrologist is recommended. Individuals who develop proteinuria should be referred to nephrology for management. Postpartum individuals whose proteinuria appears after 20 weeks' gestation should be referred to nephrology if proteinuria does not resolve within 6 weeks of delivery (Cífková, 2023).

### 4.3 Transmission risk, evaluation, and management for children

When carrier screening reveals a P/LP *COL4A3‐5* variant, screening of the reproductive partner or gamete donor should be performed to assess the chance of Alport syndrome in the child or children resulting from the pregnancy. Partner testing should consider the possibility of digenic inheritance of these genes with combinations of *COL4A3‐5* evaluated (Savige et al., 2021). Individuals with a P/LP variant in *COL4A3‐5* should be referred to genetic counseling so that the individual and their partner can be informed about the chance of transmission of the variant to a child or children, as well as the chance that existing children and other relatives may be affected. Genetic counselors may also provide information about in vitro fertilization and preimplantation genetic testing for monogenic disorders.

Because early treatment can delay the development of chronic kidney disease and kidney failure in people with Alport syndrome, children resulting from pregnancies complicated by P/LP variants in *COL4A3‐5* should undergo targeted variant analysis within the first one to 2 years of life. Children who inherit such a variant should be referred to pediatric nephrology for evaluation and management.

### 4.4 Evaluation of family members

Individuals with P/LP variants in *COL4A3–5* will frequently have relatives, including existing children, who are also heterozygous for a P/LP variant and may be candidates for treatment to delay the development of chronic kidney disease and kidney failure. Screening should include any previously untested parents, siblings, and children of the carrier. While targeted variant analysis is the ideal screening method, urinalysis will identify the great majority of affected individuals who are at chance for chronic kidney disease and kidney failure and is a reasonable alternative when targeted variant analysis is not feasible.

## 5 CONCLUSIONS

In summary, identifying P/LP variants in *COL4A3, COL4A4*, or *COL4A5* during reproductive carrier screening provides an important opportunity for early clinical intervention for Alport syndrome heterozygotes and their families. While the primary purpose of carrier screening is to assess reproductive risk, this practice serves as a gateway to uncovering significant health risks in seemingly healthy individuals. Testing needs prior informed consent and disclosure of results and requires a nuanced approach that considers individuals' perceptions and concerns. As global genomics programs continue to expand, it is imperative to fully consider the implications of identifying heterozygous variants in both reproductive and newborn screening. The example of Alport syndrome illustrates the broader value of carrier screening as a tool for both reproductive and personal health management.

## AUTHOR CONTRIBUTIONS

Vivienne Souter: Conception and design, drafting and revising. Lisa Johnson: Conception and design, drafting and revising. Emily Becraft: Conception and design, drafting and revising. Ashley Cantu‐Weinstein: Design, drafting and revising. Hossein Tabriziani: Interpretation, revising for important intellectual content. Peter Benn: Interpretation, revising for important intellectual content. Clifford E. Kashtan: Conception and design, drafting and revising. All authors approve of the final version of the manuscript and agree to be accountable for all aspects of the work.

## CONFLICT OF INTEREST STATEMENT

The authors declare the following competing interests: V.S., E.B., L.J., H.T., and A.C.‐W. are employees of Natera, Inc. with stocks or options to own stocks. P.B. is a consultant for Natera, Inc. with options to own stocks.

## Data Availability

This manuscript is a commentary in which no new data are presented, so data sharing is not applicable.
